# Mid-infrared spectroscopic screening of metabolic alterations in stress-exposed gilthead seabream (*Sparus aurata*)

**DOI:** 10.1038/s41598-020-73338-z

**Published:** 2020-10-01

**Authors:** Cláudia Raposo de Magalhães, Raquel Carrilho, Denise Schrama, Marco Cerqueira, Ana M. Rosa da Costa, Pedro M. Rodrigues

**Affiliations:** 1 Centre of Marine Sciences, CCMAR, Universidade Do Algarve, Campus de Gambelas, Edifício 7, 8005-139 Faro, Portugal; 2 Algarve Chemistry Research Centre, CIQA, Universidade Do Algarve, Campus de Gambelas, Edifício 2, 8005-139 Faro, Portugal

**Keywords:** Biochemistry, Biological techniques, Chemical biology, Systems biology

## Abstract

Stress triggers a battery of physiological responses in fish, including the activation of metabolic pathways involved in energy production, which helps the animal to cope with the adverse situation. Prolonged exposure to stressful farming conditions may induce adverse effects at the whole-animal level, impairing welfare. Fourier transform infrared (FTIR) spectroscopy is a rapid biochemical fingerprinting technique, that, combined with chemometrics, was applied to disclose the metabolic alterations in the fish liver as a result of exposure to standard stressful practices in aquaculture. Gilthead seabream (*Sparus aurata*) adults exposed to different stressors were used as model species. Spectra were preprocessed before multivariate statistical analysis. Principal components analysis (PCA) was used for pattern recognition and identification of the most discriminatory wavenumbers. Key spectral features were selected and used for classification using the k-nearest neighbour (KNN) algorithm to evaluate whether the spectral changes allowed for the reliable discrimination between experimental groups. PCA loadings suggested that major variations in the hepatic infrared spectra responsible for the discrimination between the experimental groups were due to differences in the intensity of absorption bands associated with proteins, lipids and carbohydrates. This broad-range technique can thus be useful in an exploratory approach before any targeted analysis.

## Introduction

Intensive and controlled fish production is necessary to meet the ever-increasing demand for quality protein^1^. However, intensification of production, mainly by the aquaculture industry, inevitably leads to environmental and welfare issues. Management practices often induce some level of disturbance, which can elicit a stress response in fish, may result in more severe long-term complications at the growth, reproduction, health and behaviour levels^2^. Therefore, proper monitoring of stress in fish is crucial to reduce the adverse effects of production routines. Also, a more in-depth knowledge of the physiology of fish stress becomes fundamental.

Stress in fish has been extensively studied^3^, but only recently, more modern and sensitive techniques started to be applied in this endeavour. High-throughput technologies have been gaining popularity to unveil the main changes occurring in farmed fish metabolism caused by stressful rearing conditions^4^. Among them is metabolomics, which allows for the non-selective chemical analysis of metabolites in a given biological system^5^. Metabolomics in fish research has been focused mainly on the environmental impacts on fish health^6,7^ and welfare^8,9^. Metabolic fingerprinting is one common approach in metabolomics, often of comparative nature, that can provide qualitative information on the metabolic alterations caused by biotic or abiotic factors^10^. Among different techniques, Fourier transform-infrared (FTIR) spectroscopy, a form of vibrational spectroscopy, is one common analytical platform used in metabolic fingerprinting. It has been successfully applied to differentiate functional biochemical groups in the liver of fish exposed to different rearing conditions^11,12^, to determine the changes caused by spoilage in gilthead seabream^13^ and salmon^14^, and to identify alterations provoked by toxic chemicals, in rainbow trout^15,16^, rohu^17,18^, Mozambique tilapia^19^ and catfish^20^. Furthermore, FTIR spectroscopy was also applied to assess cod liver oil quality^21^ and characterize hake lipids and lipid changes during frozen storage^22^. Although the use of FTIR spectroscopy in fish research is still in its infancy, these studies with such diverse contexts underline the applicability of this technique to study different fish tissues.

Vibrational spectroscopy is based on the ability of certain compounds, presenting covalent bonds, to selectively absorb unique frequencies of electromagnetic radiation, exciting the molecule to a higher vibrational state. Each chemical bond can vibrate in numerous ways, and each vibration is called a vibrational mode (e.g., stretching or bending). This absorption of energy by the vibrating chemical bond results in an infrared spectrum. The most commonly used techniques based on vibrational energy are Raman and infrared (IR) spectroscopies^23^.

FTIR spectroscopy is a fast and relatively simple technique, with a low-cost value regarding consumables^24^ and requiring a small amount of sample^25^. First, an interferogram is collected from a sample signal using an interferometer. Then a Fourier transform (a mathematical algorithm) is applied to the raw data (interferogram) to obtain the actual infrared spectrum, in the mid-infrared region^10^. Hence, this technique allows to analyse changes in band positions, widths and intensities to obtain information on the metabolic changes with alterations in main compounds, such as lipids, proteins and carbohydrates^26^.

No reference has been found in the literature regarding the use of FTIR spectroscopy to investigate the metabolic alterations in farmed fish induced by everyday aquaculture production stressors. However, previous studies on the effects of stressful conditions in algae^27^, yeast^28^, bacteria^29^ and fish toxicology^19^ demonstrate the potential of its application in this field. One great advantage of this spectroscopic technique is its ability to provide a broad outlook on fish metabolism without any preconceptions. Additionally, its holistic nature can offer a global overview of the classes of biochemical compounds responding directly to external stimuli, which can be extremely useful before any targeted and more accurate analysis.

In the present work, gilthead seabream (*Sparus aurata*) adults, a widely cultured species in the European aquaculture, were submitted to three different stressful rearing conditions, namely overcrowding, net handling and hypoxia. These stressors were demonstrated before to induce significant changes in the levels of specific metabolites, known to be associated with the physiological stress response in fish, and stress-related proteins, in the blood plasma of farmed gilthead seabream (*Sparus aurata*)^4^. In this work, potential metabolic changes were investigated in the liver of the challenged fish, since this organ plays a key role in the metabolic responses triggered by the stress response, mainly in the supply of energy for the animal to cope with adverse situations^2^. FTIR spectra were obtained from liver tissue samples and different chemometric techniques were employed for the efficient processing of the high-dimensional datasets generated. This untargeted approach aimed to explore the potential of this technique to screen for spectral changes in the fish liver’s metabolic profile and provide a broad overview of the alterations caused by specific farming conditions.

## Results and discussion

In this study, gilthead seabream adults were submitted to three different rearing conditions in separate trials. The chosen stressors are commonly encountered in aquaculture production routines: overcrowding (OC), net handling (NET) and hypoxia (HYP). The livers of stressor-exposed fish were compared to those of control fish using FTIR spectroscopy^30^, which allows to perform a rapid screening of the biological system under investigation and thus to detect unforeseen metabolic alterations. Hence, this analysis generates a ‘holistic’ overview of the potential changes in major functional groups retrieved from the challenged fish, making FTIR spectroscopy a suitable technique to be used prior to any targeted analysis^26^. To the best of our knowledge, this is a pioneering work using FTIR spectroscopy to screen for the effects of stressors associated with standard aquaculture practices in the metabolic profile of farmed fish.

### FTIR spectra of gilthead seabream liver submitted to stressful conditions

**Figure 1:**
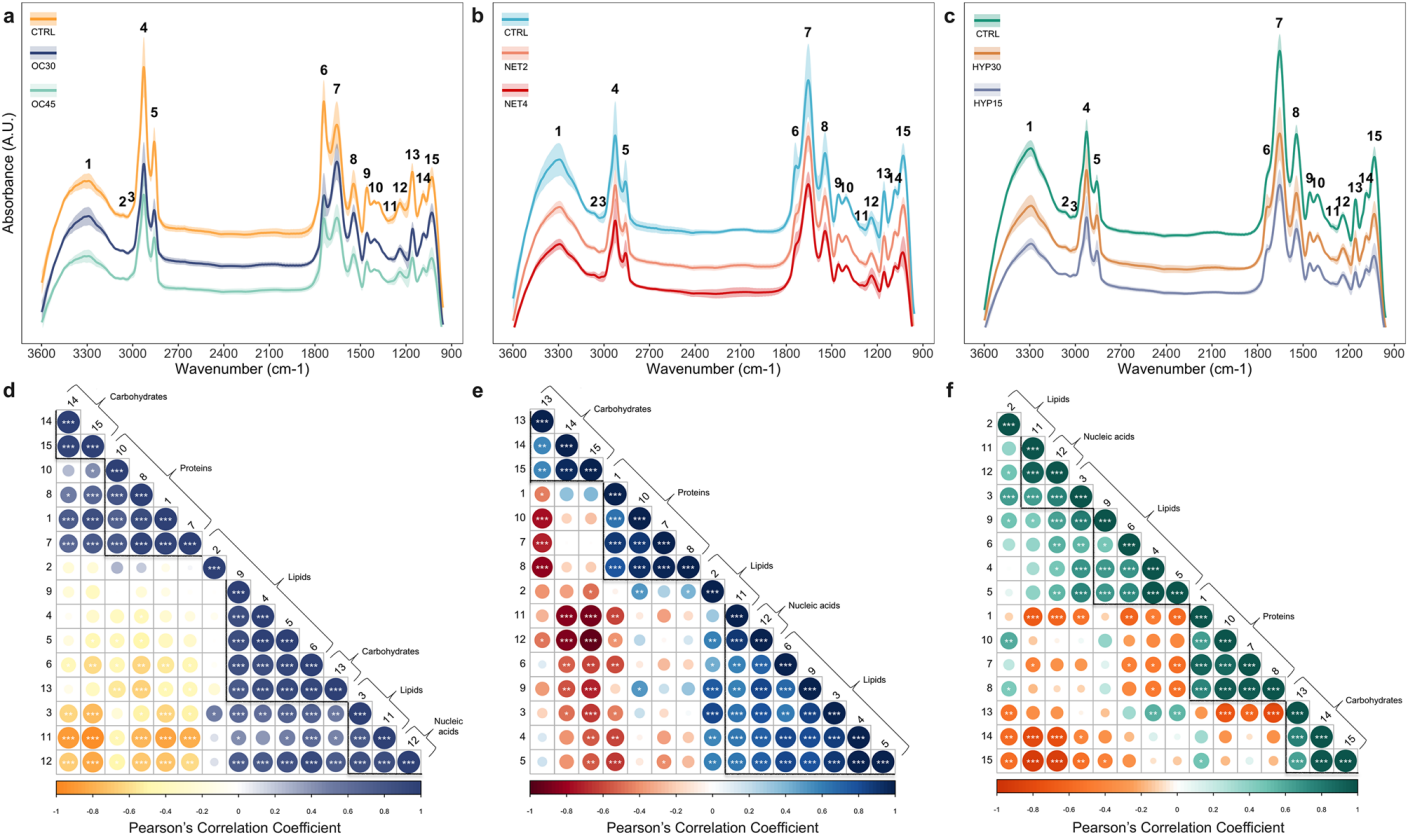
Fourier transformed infrared (FTIR) spectra of gilthead seabream (*Sparus aurata*) liver submitted to three different stressful rearing conditions (overcrowding, net handling and hypoxia) and Pearson’s correlation coefficient matrices comparing the assigned bands of the spectra. (**a**–**c**) FTIR spectra, for each treatment, are shown as absorbance values (in arbitrary units (A.U.)) of 8 averaged spectra (solid line) ± standard deviation (shaded ribbon). For easier readability, mean spectra were offset along the absorbance axis. Numbers indicate the bands assigned to biomolecules, listed in Table 1. Plots in each row are prepared with the same vertical scale. (**d**–**f**) Plots are ordered by hierarchical clustering with complete linkage. Numbers indicate the bands assigned to biomolecules, following the same convention as Table 1. Thicker lines represent clusters. The degree of pairwise correlation concerning Pearson's correlation coefficient is displayed by the colour gradient and dot size, while the colours define the signal of the correlation (positive or negative). The significance of the correlation is indicated by the label “*” inside the dots (*0.05 < *P* < 0.01, **0.01 < *P* < 0.001, *** *P* > 0.001).

Overall, the FTIR spectra of the fish liver from the three trials showed the typical complex metabolic patterns with several overlapping bands observed mainly at two frequency regions: 3600–2800 cm^−1^ and 1800–950 cm^−1^. Only the spectral region between 3600 and 950 cm^−1^ was used for further analysis as both the head and end of the spectra showed excessive noise (see Supplementary Fig. S1). For each experimental treatment, 18 spectra were recorded. The acquisition of spectra in transmission mode can be affected by several factors such as sample uniformity and homogeneity, and thickness of the KBr pellet^31^. Therefore, spectra were pre-processed before multivariate statistical analyses. Variations were thus minimized by detrending, which also removed the effects of baseline shifts, and the noise reduced with Savitzky–Golay filtering. Raw and treated spectra are shown in Supplementary Fig. S1. The total spectrum was characterized by 15 bands (Fig. 1a–c) which were assigned to specific vibrational modes, functional groups and biochemical compounds based on the correlation analysis performed and similar biological systems described in the literature^11,12,18,22,32,33^ (Table 1). Different chemometric techniques were employed to discriminate the different spectral regions and experimental treatments.

**Table 1:** Tentative assignment of spectral bands to molecular vibrations of functional groups and biochemical compounds, based on similar biological systems described in the literature^11,12,18,22,32,33^.

Band	Wavenumber (cm^−1^)	Vibrational modes and functional groups	Main biochemical compounds	Other biochemical compounds
1	3315–3290	N–H stretching of amides (Amide A) O–H stretching of polysaccharides	Proteins	Carbohydrates
2	3065	Olefinic = C–H stretching	Unsaturated fatty acids	Aromatics
3	3010	Olefinic = C–H stretching	Unsaturated fatty acids	Aromatics
4	2926	CH_2_, CH_3_ asymmetric stretching	Saturated lipids	Proteins, carbohydrates, nucleic acids
5	2858	CH_2_, CH_3_ symmetric stretching	Saturated lipids	Proteins, carbohydrates, nucleic acids
6	1750–1739	C=O stretching of esters and aldehydes	Triglycerides, cholesterol esters	Lipids, phospholipids
7	1655	C=O stretching of amides (Amide I) C=C stretching of unsaturated hydrocarbons	Proteins	Unsaturated fatty acids
8	1541	N–H bending and C–N stretching of amides (amide II) C=C stretching of aromatic hydrocarbons	Proteins	Aromatics
9	1455	CH_2_ symmetric and asymmetric bending	Lipids	Proteins
10	1415–1395	COO^−^ symmetric stretching	Amino acids and fatty acids	Other carboxylates
11	1305	Olefinic C–H bending P=O stretching in phosphates	Unsaturated fatty acids	Alcohols, aromatic amino acids organic phosphates, carboxylates
12	1240	PO^−^_2_ asymmetric stretching	Nucleic acids	Phospholipids
13	1155	CO–O–C asymmetric stretching of esters and glycogen = C–H bending in aromatics	Phospholipids and Carbohydrates	Aromatics, cholesterol esters
14	1085	C–O stretching of glycogen PO^−^_2_ symmetric stretching	Carbohydrates	Phospholipids
15	1045–1025	O stretching of glycogen	Carbohydrates	

### Correlation analysis

Correlation analysis of the 15 bands assigned to specific biomolecules (Table 1) revealed 4 major clusters in the OC trial, with significant Pearson’s correlation coefficients greater than 0.71 for all band pairs (Fig. 1d). Cluster 1, counting from the top of the matrix, (*r* > 0.95, *P* < 0.001) is comprised exclusively of carbohydrate-like bands, whereas cluster 2 (*r* > 0.71, *P* < 0.001) of bands assigned to proteins. The third cluster (*r* > 0.76, *P* < 0.001) consisted of 4 bands assigned to lipids, with band nº 13 assigned to carbohydrates and phospholipids. The fourth (*r* > 0.77, *P* < 0.001) is a cluster of 3 bands, of which two are assigned to unsaturated fatty acids and the third to nucleic acids and phospholipids. For the NET trial, the correlation analysis originated 3 very well-defined clusters, with significant Pearson’s correlation coefficients greater than 0.58 for all band pairs (Fig. 1e). The first cluster (*r* > 0.58, *P* < 0.001) grouped 3 bands assigned to carbohydrates, cluster 2 (*r* > 0.66, *P* < 0.001) consisted of 4 bands assigned exclusively to proteins, and cluster 3 (*r* > 0.58, *P* < 0.001) was formed mainly by bands assigned to lipids and band nº 12, which was assigned to nucleic acids and phospholipids. In the HYP trial case, the correlation analysis grouped the bands in 4 clusters, with significant Pearson’s correlation coefficients greater than 0.47 for all band pairs (Fig. 1f). Clusters 1 (*r* > 0.65, *P* < 0.001) and 2 (*r* > 0.47, *P* < 0.02) were mainly formed by bands assigned to lipids and band nº 12, which was assigned to nucleic acids and phospholipids. Similarly to the NET trial, clusters 3 (*r* > 0.66, *P* < 0.001) and 4 (*r* > 0.63 *P* < 0.001) consisted of bands assigned to carbohydrates and proteins, respectively. Band nº 2 presented low correlations in the OC trial. Absorption band nº 13 can be attributable to either carbohydrate and/or phospholipids. However, the correlation analysis suggests that in the OC trial, it is more likely to represent changes in the phospholipids’ content. Contrarily, in the NET and HYP trials, it seems to correspond to carbohydrates.

### Principal component analysis

**Figure 2:**
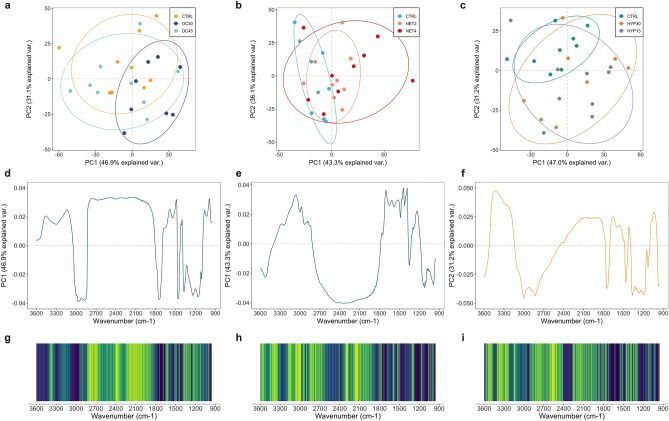
Principal component analysis (PCA) on the Fourier transformed infrared spectra collected from the livers of gilthead seabream (*Sparus aurata*) submitted to three different stressful rearing conditions (overcrowding, net handling and hypoxia). (**a**–**c**) Score scatter plots on PC1 and PC2 computed for each trial with the 3600–950 cm^−1^ spectral range. Each point represents the projection of one spectrum, and each treatment is identified by a unique colour, as indicated in the legend. Percentages indicate the proportions of explained variance. Ellipses represent an 80% probability of samples being within the shape. (**d**–**f**) Principal component loadings along the corresponding wavenumber for each trial. (**g**–**i**) Ranking of the spectral features according to the SVM-RFE method for feature selection, along the wavenumber range of 3600–950 cm^−1^. Most well classified features in the ranking are shown in dark blue, while least important features are coloured in yellow.

Principal component analysis (PCA) is an unsupervised method, with no a priori knowledge of experimental structure, primarily used to reduce the dimension of the feature space, detect structural relationships between variables and find potential clusters of observations. The original correlated variables are transformed into a set of orthogonal uncorrelated variables, linear combinations of the first ones^34^. In this study, PCA was employed for exploratory analysis. A score scatter plot was generated, for each trial, with the projection of the samples onto the first two principal components (PCs), which accounted for 78%, 79.4% and 78.2% of the total variability of the data from OC, NET and HYP trials, respectively (Fig. 2a–c). The analysis of the samples’ grouping in the score plots suggests that the separation between the corresponding control samples and those belonging to the OC30 (OC trial) and NET4 groups (NET trial) occurred along the PC1 axis. At the same time, PC2 appears to be responsible for the dissimilarities between the control group and the HYP15 group (HYP trial). Observations from groups OC45, NET2 and HYP30 are largely overlapped with the other experimental groups. Loading plots in Fig. 2d–f, illustrate the weight of each of the original variables (wavenumbers) on the PCs, and thus, the contribution of each spectral feature to discriminate the mentioned pairs of treatments. Positive loading values in the OC30 and NET4 plots (Fig. 2d,e) indicate a higher concentration, in challenged fish, of the biomolecules corresponding to the indicated spectral ranges. The inverse is verified for the HYP trial loading plot (Fig. 2f). The various lobes observed in the plots with high absolute loading values suggest that the separation of the two most distinct groups, observed in the score plots, is based on different spectral regions. The PC1-loadings belonging to the OC trial (Fig. 2d), revealed the strongest negative loadings in the 3020–2800 cm^−1^, 1740 cm^−1^ and 1450 cm^−1^ spectral regions, which correspond to vibrational bands highly associated to lipids (bands nº 3, 4, 5, 6 and 9), and the strongest positive loadings in the regions 1600–1500 cm^−1^ and 1030 cm^−1^ (bands nº 7, 8 and 15), which were attributed to proteins and carbohydrates’ bands, respectively. The NET trial’s PC1-loading plot (Fig. 2e) shows the main positive loading peaks at 3060, 1710, 1450 and 1400 cm^−1^ (bands nº 2, 9 and 10), which correspond to bands assigned to vibrational modes of proteins and lipids, and the higher negative loading peaks around 1150 and 1030 cm^−1^ (bands nº 13 and 15), corresponding to the carbohydrate characteristic region. The PC2-loadings of the HYP trial (Fig. 2f) show the most intense positive peak in the region between 3300 and 3400 cm^−1^, and the strongest negative loadings at 3000–2800, 1740 and 1350–1150 cm^−1^, corresponding mainly to lipid-assigned bands (bands nº 3, 4, 5, 6, 11, 12 and 13). These potential changes in these absorption bands appear to be all correlated and suggest a metabolic reprogramming in the fish system to deal with the increase of energy demand during adverse situations. The plasma levels of specific metabolites associated with the physiological response to stress were assessed in these fish in a previous study and published elsewhere^4^. Activation of the HPI-axis was previously suggested and supports the hypothesis of a potential metabolic reprogramming to deal with the stressors that fish were exposed to^4^. When fish is exposed to a challenging situation, a physiological response initiates to compensate and/or adapt to the new situation^2^. When the coping capacity is surpassed, the so-called stress response mechanism is initiated by the rapid release of catecholamines into the bloodstream, followed by the delayed response of cortisol, which further widespreads effects on various tissues^35,36^. Carbohydrates are essential and rapid sources of energy for fish in stressful situations^3^. A potential hepatic carbohydrate increment in fish from OC30 group suggests the constant activation of the major gluconeogenic pathway by the chronic cortisol release. This leads to the synthesis of glucose in the liver, which, if not used or exported, can be stored in the form of glycogen (glycogenesis)^37^. Contrarily, the suggested decrease in hepatic carbohydrate content in NET4 fish is consistent with the stressor’s physically more intense nature. This reduction suggests the use of glycogen stores, by glycogenolysis, to synthesize glucose, and its immediate uptake, for energy production, or outflow^2,38^. These results are consistent with previously reported plasma glucose levels for these fish^4^. Proteins and amino acids are essential non-carbohydrate substrates for the gluconeogenesis and have been described as hepatic energy fuels in fish under different stressful conditions^37^. Results suggest that the protein and amino acid contents increased in the liver of OC30 and NET4 fish, which can be indicative of a cortisol-mediated increased proteolytic activity and consequent mobilization of amino acids to the liver to be used as gluconeogenesis precursors. To some extent, this pattern reinforces the hypothesis of the activation of this pathway. Nonetheless, increased protein content can also be explained by a higher protein turnover and/or synthesis of proteins involved in gluconeogenesis^39^. Finally, the OC and HYP trials PC-loadings suggested that the separation between control and OC30/HYP15 groups might be due, mainly, to potential differences in the spectral bands associated to vibrational modes of lipids. Lipid metabolism in fish is also modulated by cortisol^37^. Glycerol, resulting from the catabolism of triacylglycerols is a suitable precursor for gluconeogenesis, while fatty acids are used as sources of energy in peripheral tissues^40^. These differences suggest a cortisol-mediated activation of hepatic lipolysis and the posterior use of the lipids as substrates for gluconeogenesis and/or mobilization to other tissues^37^. Other studies with fish exposed to high stocking densities report a reduction in hepatic lipid content and/or increased exportation of fatty acids into the bloodstream^41,42^. Previous studies on different fish species also report a mobilization of lipids to the liver during exposure to prolonged hypoxia^43,44^. More targeted hypothesis-driven approaches will be interesting to confirm the effects of these farming conditions on the described metabolic pathways.

### Feature selection and k-nearest neighbour classification analysis

In order to assess if the spectral features suggested to be responsible for the separation between the groups in the score plots generated by the unsupervised PCA were indeed discriminatory, a supervised classification analysis was performed. A full infrared spectrum contains hundreds or thousands of variables, and the neighbouring wavenumbers are always collinear. Hence, selecting the most discriminatory wavenumbers and discarding the uninformative ones can improve the accuracy and robustness of multivariate analyses and classification models^45^. Spectral feature selection was achieved by a support vector machine based on recursive feature elimination (SVM-RFE)^46^. Compared with other feature selection methods, SVM-RFE is a scalable and efficient wrapper method. Firstly, linear SVM is trained on the initial set of features while assigning weights (w) to each one, and then RFE selects feature subsets by recursively considering smaller subsets of features at each time^47^. Features were incrementally selected 5–30 features with five steps, according to the importance of ranking, as input data to the classifier.

**Figure 3:**
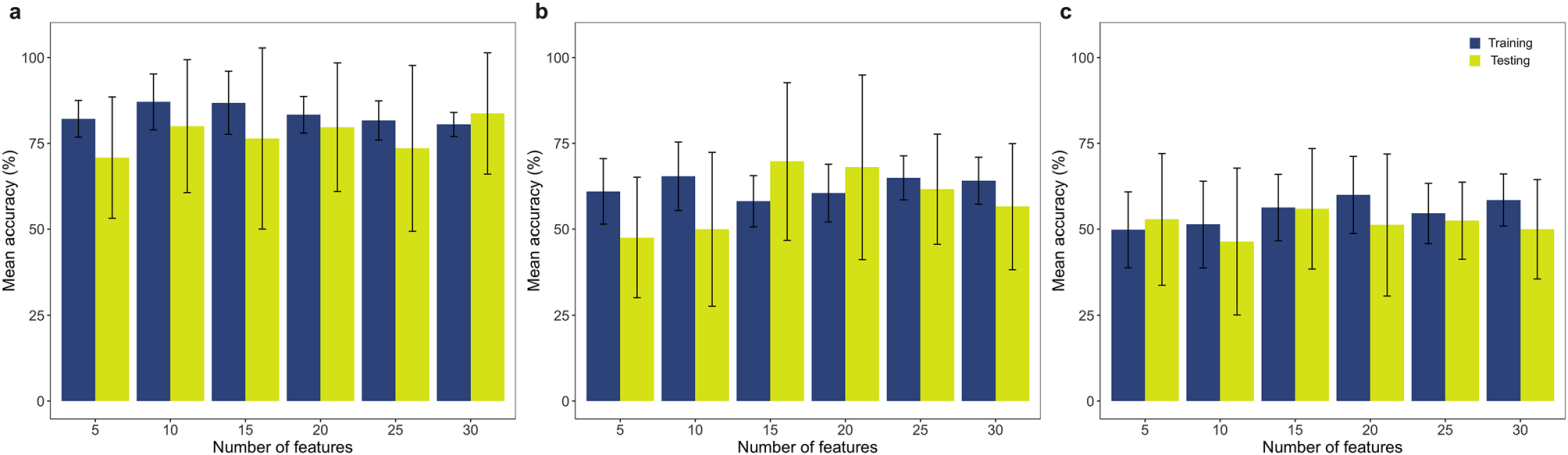
Classification analysis performed by the k-nearest neighbour (KNN) algorithm on the Fourier transformed infrared spectra collected from the livers of gilthead seabream (*Sparus aurata*) submitted to three different stressful rearing conditions ((**a**) OC trial, (**b**) NET trial, (**c**) HYP trial). Predictive performance of the models are presented as mean classification accuracy (%) of training and testing sets for each subset of selected features by SVM-RFE. Error bars represent the standard deviation obtained by tenfold cross validation of the initial data splitting.

Plots displaying the importance of each spectral feature in the ranking are shown along with the wavenumber range of 3600–950 cm^−1^ in Fig. 2g–i for comparison with the loadings generated by PCA . Selection of the most informative wavenumbers is generally in accordance with PCA loadings, except for the regions between 3200–3000 and ~ 3300 cm^−1^, in the spectra from NET and HYP trials. The supervised classification analysis with the KNN algorithm was carried out for each trial, using only two out of the three classes: “control” and the experimental group that presented the most clear separation in the corresponding score plots (i.e. OC30, NET4 and HYP15, in the case of OC, NET and HYP trials, respectively). The KNN algorithm is a non-parametric supervised classification method that allows categorizing unknown samples based on multivariate proximity to other samples of pre-assigned classes (majority voting)^48^. The unknown sample’s identity is based on the class of the nearest known samples, where each class represents an experimental group. In this process, 6 models were built, for each trial, with different subsets of features. The optimal number of neighbours (k) was calculated by LOOCV, where one point in the data set is set as the test data, and the remaining points are set as the training data. The classifier’s performance is shown in Fig. 3 and presented as the mean prediction accuracy ± standard deviation for the train and test datasets along the different subsets of selected features. The highest accuracy values were obtained for the classification analysis of the OC groups, with a subset of 10 spectral features (87% and 80% for the train and test datasets). Overall, the classification models for the NET4 and HYP15 trials had a satisfactory/poor performance in the discrimination of the experimental groups, which appears to be due, mainly, to high variability of biological responses. Increasing the number of observations per group could potentially improve the classification models’ performance and predictive ability.

## Conclusions

In light of the present findings, FTIR spectroscopy coupled with chemometric analysis of spectral data presents itself as an exploratory starting approach for the collective screening of alterations in the metabolism of proteins, lipids and carbohydrates simultaneously. Moreover, its holistic nature makes this technique a suitable analysis to be employed prior to any targeted approach. This study showed the main spectral differences in the liver of gilthead seabream exposed to high rearing densities (30 kg/m^3^), net handled four times a week and exposed to low levels of saturated oxygen (15%) when compared to the control groups. These alterations point towards a potential activation of the fish stress response and a consequent global rearrangement of the metabolism of the main biochemical compounds. Finally, a supervised classification analysis demonstrated that the ten most informative wavenumbers could discriminate between control and crowded fish with relatively reasonable classification accuracy. However, increasing the number of samples of the experimental treatments could benefit the overall predictions. This work introduces FTIR spectroscopy in fish stress research as a rapid broad-range tool to extract untargeted information regarding alterations on the hepatic IR spectra of fish exposed to challenging farming conditions. Validation analysis will greatly contribute to link these spectral changes to the fish liver biochemistry and potential alterations in specific biochemical compounds and metabolic pathways involved in the fish physiological stress response.

## Methods

### Experimental design and sampling

The experiments were conducted at the Ramalhete Experimental Research Station of the Centre of Marine Sciences (CCMAR), in Faro, Portugal. For each trial, nine homogeneous groups of gilthead seabream (*Sparus aurata*) adults (supplied by a commercial fish farm—Maresa, Mariscos de Estero S.A., Huelva, Spain) were randomly stocked in indoor 500 L conical fiberglass tanks supplied with flow-through seawater. Each trial was conducted in a different period. Throughout the trials, the physicochemical parameters varied within a natural regime (natural photoperiod, temperature at 13.4 ± 2.2 °C, salinity at 34.7 ± 0.8‰ and dissolved oxygen level above 5 mg L^−1^). Fish were fed by hand once daily, in the morning, according to the fish initial body weight and the water temperature, with commercially available 6 mm feed (AquaSoja, Sorgal, S.A., Ovar, Portugal), manufactured according to the species’ nutritional requirements.

Following a 2-week acclimation period, three stress trials were established: OC—Overcrowding, NET—Net Handling, and HYP—Hypoxia. Each tank, with an initial rearing density of 10 kg/m^3^ (except for the high-stocking groups), was randomly allocated to one of the three treatments, in triplicate. The OC trial lasted for 54 days, and the fish, with an initial body weight of 372.33 ± 6.55 g, were subjected to high stocking densities over the entire experimental period, by increasing the number of fish in the tanks. Two intensities were tested, having as experimental groups: OC_CTRL_—10 kg/m^3^, OC_30_—30 kg/m^3^ and OC_45_—45 kg/m^3^. For the NET trial, fish, with initial body weights of 375.69 ± 11.88 g, were stressed for 45 days. Specific nets were designed, fitted inside the tanks and lifted to air-expose the fish for 1 min. The experimental groups were established according to the number of times that the fish were lifted: NET_CTRL_—undisturbed fish (the net was likewise fitted inside the tanks but not lifted), NET_2_—fish air-exposed twice a week, and NET_4_—fish air-exposed four-times a week. In the HYP trial, fish, with an initial body weight of 397.99 ± 16.56 g, experienced low levels of saturated oxygen over 48 h. Nitrogen was injected in the water according to the experimental groups’ requirements: HYP_CTRL_—100% saturated oxygen, HYP_30_—30% saturated oxygen, and HYP_15_—15% saturated oxygen. Data regarding zootechnical parameters were previously published by the authors^4^.

At the end of each experimental period, three fish per tank (n = 9 per treatment) were lethally anaesthetized using tricaine methanesulfonate (MS-222; Sigma Aldrich, St. Louis, Missouri, USA). The livers of the sampled fish were collected for FTIR analysis and immediately frozen at − 80 °C. Before harvesting, fish were starved for 48 h to clean the digestive tract.

This study was approved by the ORBEA Animal Welfare Committee of CCMAR and the Portuguese National Authority for the Animal Health (DGAV) on August 26th, 2019. The experiment described was conducted in accordance with the European guidelines on the protection of animals used for scientific purposes (Directive 2010/63/EU) and the Portuguese legislation for the use of laboratory animals, under a “Group-1” license (permit number 0420/000/000-n.99–09/11/2009) from the Veterinary Medicine Directorate, the competent Portuguese authority for the protection of animals, Ministry of Agriculture, Rural Development and Fisheries, Portugal and following category C FELASA recommendations.

### Sample preparation and FTIR spectroscopy analysis

Prior to FTIR analysis, liver samples were lyophilized for 48 h in a FreeZone 6 L Freeze Dry System (LabConco, Kansas City, MO, USA), to prevent peaks derived from O–H molecular vibrations of water molecules. Lyophilized samples were ground in an agate mortar and pestle and blended with potassium bromide (Sigma Aldrich, St. Louis, MO, USA) until homogeneous, in a 1:3 ratio. The mixture was then placed in an evacuated die (13 mm diameter) and pressed (6 × 10^6^ Pa) for 2 min to obtain approximately a 1 mm-thick translucent pellet, which was then used for analysis by solid-phase transmissive FTIR spectroscopy.

Two individual FTIR spectra were acquired per biological sample (at distinct points of the pellet), in the transmission mode, using a FTIR Spectrophotometer (TENSOR 27 series, Bruker Optik GmbH, Ettlingen, Germany) controlled by the OPUS software (v5.5, Bruker GmbH). To enhance the signal-to-noise ratio (SNR), interferograms were averaged for 32 scans at 4 cm^−1^ resolution, over the middle-infrared wavenumber range of 4000–600 cm^−1^, to obtain a single spectrum. Atypical observations (extreme outliers potentially due to technical errors during sample preparation and/or spectral acquisition) were immediately inspected and repeated if needed. Transmittance spectra generated were exported for further analysis.

### Spectral preprocessing

The .dpt files from OPUS were imported into R v3.5.3^49^ for MacOSX where all data preprocessing, univariate and multivariate statistical analyses were performed. Each spectrum was corrected with a straight line fitted between 2410 and 2270 cm^−1^ to compensate for the atmospheric CO_2_ peaks, converted from transmittance to absorbance (*A* = log_10_ 1/*T*) and truncated to the spectral region of interest between 3600 and 950 cm^−1^. De-trending was applied for baseline correction and standard normal variate (SNV) transformation, followed by smoothing over 25 points with the Savitzky–Golay filter. All preprocessing techniques were applied using the *prospectr* package^50^. Outlier spectra detection was carried out by a robust principal component analysis (PCA) through the projection pursuit approach and using the GRID algorithm^51^. One bad leverage point and one orthogonal outlier were removed from the corresponding datasets (see Supplementary Fig. S2). The functions PCAgrid and pcaDiagplot used for this analysis belonged to the *pcaPP* and *chemometrics* packages^52,53^, respectively. Data points considered outliers were removed from further analyses. FTIR spectra from technical replicates were averaged by arithmetic mean. Determination of FTIR band positions (wavenumber (cm^−1^)) was performed, per averaged spectrum, according to the centre of weight, using the peak-picking function of the Essential FTIR software (free trial version, Operant LLC, Madison, WI, USA). Tentative assignments of spectral features to classes of biochemical compounds are described in Table 1.

### Multivariate statistical analyses

For statistical analyses, FTIR wavenumbers and absorbance values were treated as independent and dependent variables, respectively. Each trial was analysed separately. To assess metabolic patterns between assigned bands, the Pearson’s correlation coefficient and its significance were calculated for the FTIR band matrix using the rcorr function, from package *Hmisc*^54^*.* The correlation matrix was illustrated through a correlogram using the function corrplot from package *corrplot*^55^. Detection of structural relationships between variables and pattern recognition was carried out through an exploratory PCA, using the standard prcomp R function in the auto-scaled matrices. The ordination diagram was generated for each trial with the assigned object scores relative to each principal component (PC1 and PC2). The loadings corresponding to the most discriminative PCs were plotted to visualise and identify the most informative spectral features. Finally, a supervised classification analysis was performed to investigate whether the spectral changes allowed for the reliable discrimination between experimental groups. Feature selection was adopted to retain relevant information and deduct irrelevant information. This was achieved by support vector machine based on recursive feature elimination (SVM-RFE) and the top-ranked spectral features used as inputs for the k-nearest neighbour (KNN) classifier. Data splitting into training (70%) and testing (30%) sets was tenfold cross validated to ensure that every observation was incorporated into the testing set. Each training set was normalized by centering and scaling, and the parameters used to normalize the test set. Every training dataset was then used to train a model, and re-sampling was achieved by leave-one-out cross-validation (LOOCV). External validation was finally performed on a testing dataset, with control as the positive class. The optimal number of neighbours (k) was also determined by inner LOOCV on the training sets, using accuracy as the parameter for selection, from 1 to 5, with a step of 2. Feature selection was performed using the package sigFeature^56^, and the classification analysis using the functions trainControl and train from the *caret* package^57^. Categorization was predicted by the function predict from the same package.

## Supplementary information

Supplementary Information.
