# Supplementary material for: Mid-infrared spectroscopic screening of metabolic alterations in stress-exposed gilthead seabream (Sparus aurata)
Source: Sci Rep. 2020 Oct 1;10:16343. doi: 10.1038/s41598-020-73338-z (PMC7529800; doi:10.1038/s41598-020-73338-z)
Supplement: Supplementary file 1 — Supplementary Information. [file 41598_2020_73338_MOESM1_ESM.pdf]

Mid-infrared spectroscopic screening of metabolic alterations in stress-exposed Gilthead seabream (*Sparus aurata*)

Cláudia Raposo de Magalhães[1,a], Raquel Carrilho[1,a], Denise Schrama[1], Marco Cerqueira[1], Ana M. Rosa da Costa[2], Pedro Rodrigues[1,*]

[1] Centre of Marine Sciences, CCMAR, Universidade do Algarve, Campus de Gambelas, Edifício 7, 8005-139 Faro, Portugal

[2] Algarve Chemistry Research Centre, CIQA, Universidade do Algarve, Campus de Gambelas, Edifício 2, 8005-139 Faro, Portugal

[a] Both authors contributed equally to this work

* Corresponding author: pmrodrig@ualg.pt

Supplementary Information

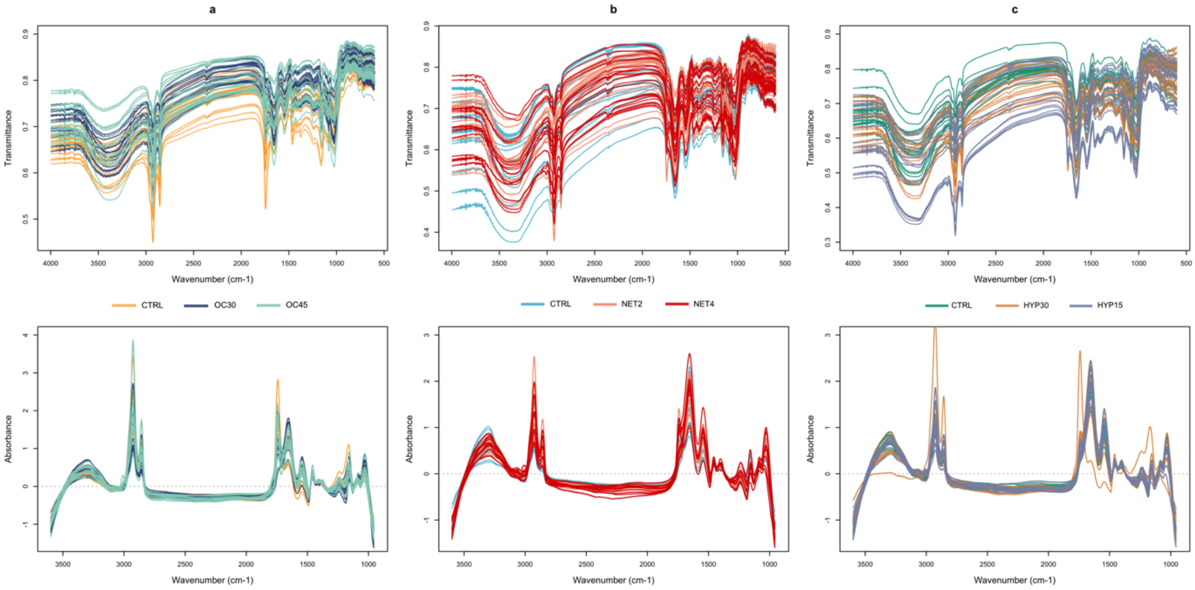

Supplementary Figure S1 - Fourier transform infrared (FTIR) spectra (n = 18 per experimental condition) of Gilthead seabream (*Sparus aurata*) liver submitted to three different stressful rearing conditions (**(a)** – OC trial, **(b)** – NET trial, **(c)** – HYP trial). Raw transmittance spectra are represented in the first row, while processed absorbance spectra, used for multivariate and univariate analyses, are displayed in the second row.

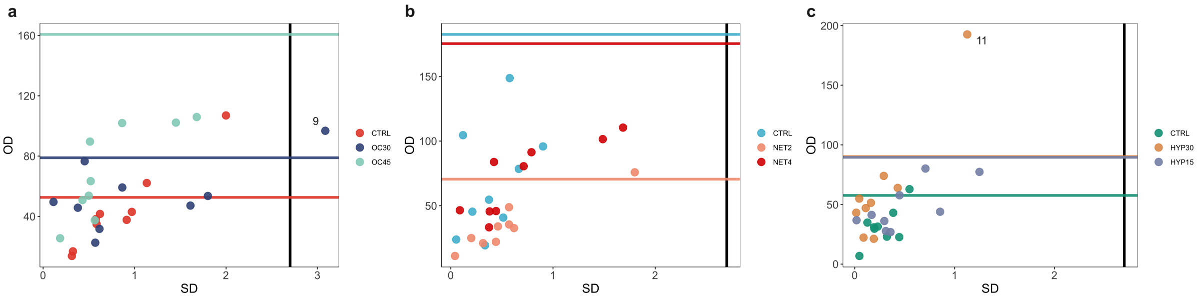

Supplementary Figure S2 – Multivariate outlier detection on the fourier transform infrared (FTIR) spectra of Gilthead seabream (*Sparus aurata*) liver submitted to three different stressful rearing conditions (**(a)** – OC trial, **(b)** – NET trial, **(c)** – HYP trial). A robust principal component analysis was performed through the projection pursuit method using the GRID algorithm. Bad leverage points and orthogonal outliers which proved to be a some kind of measurement error were eliminated from the datasets, here represented by the corresponding sample number.
